# Selective Dry Cow Therapy in Modern Dairy Management: Balancing Udder Health and Antimicrobial Stewardship

**DOI:** 10.3390/vetsci12060580

**Published:** 2025-06-12

**Authors:** Ionela Delia Ut, Daniel Ionut Berean, Liviu Marian Bogdan, Simona Ciupe, Sidonia Gog Bogdan

**Affiliations:** 1 Department of Reproduction, Faculty of Veterinary Medicine, University of Agricultural Sciences and Veterinary Medicine Cluj-Napoca, Calea Manastur 3-–5, 400372 Cluj-Napoca, Romania; ionela-delia.ut@student.usamvcluj.ro (I.D.U.); liviu.bogdan@usamvcluj.ro (L.M.B.); simona.ciupe@usamvcluj.ro (S.C.); 2 Department of Surgery and ATI, Faculty of Veterinary Medicine, University of Agricultural Sciences and Veterinary Medicine, 400372 Cluj-Napoca, Romania; sidonia.bogdan@usamvcluj.ro

**Keywords:** dry cow therapy, mastitis control, antibiotic resistance, dairy cows, udder health

## Abstract

In recent decades, Blanket Dry Cow Therapy (BDCT) has been regarded as a cornerstone strategy for the control of mastitis in dairy cows during the dry period. However, concerns regarding the rising incidence of antibiotic resistance and the associated zoonotic risks have prompted a paradigm shift, leading to intensified research into alternative management approaches. In response, many countries have adopted a more targeted approach, known as Selective Dry Cow Therapy (SDCT), which focuses on the therapeutic use of antibiotics, administered only to cows or quarters that are either infected or at high risk of infection during the dry period. This review provides a comprehensive synthesis of the scientific literature regarding the main methods for selecting animals for SDCT, the impact of this strategy on udder health, milk production, farm economics, and antibiotic consumption, as well as the factors that may influence its effectiveness. Over time, a range of methods have been developed to identify infected animals, including bacteriological culture, somatic cell count (SCC), differential somatic cell count (DSCC), and the California Mastitis Test (CMT), which are often used alone or in combination with clinical mastitis history and/or parity. Among these methods, SCC has proven to be the most economically viable and best suited for practical use, while its combination with DSCC has been shown to significantly enhance diagnostic accuracy. According to the studies reviewed, SDCT is a safe and effective strategy for maintaining udder health and farm profitability, as long as infected cows are accurately identified, and internal teat sealants are used in quarters not treated with antibiotics during the dry period. However, since udder health is influenced by herd characteristics, management practices, and regional pathogens, the findings cannot be universally applied and must be adapted to each herd’s specific conditions.

## Simple Summary

Mastitis is a widespread and costly disease in dairy cows, typically managed during the dry period using Blanket Dry Cow Therapy (BDCT), where all cows receive antibiotics. However, concerns about antibiotic resistance and the potential spread of zoonotic diseases have led to the development of a more precise strategy known as Selective Dry Cow Therapy (SDCT). Unlike BDCT, SDCT targets only cows or quarters that are infected or at high risk, reducing overall antibiotic use while preserving udder health and farm productivity. This review aims to provide a comprehensive analysis of global SDCT adoption trends, evaluating its impact on animal health, economic outcomes, and the risks associated with antimicrobial resistance (AMR), a critical global challenge, to guide effective, evidence-based implementation.

## 1. Introduction

The dry period represents a crucial phase for the maintenance of udder health and the optimization of subsequent lactation performance. It is characterized by a heightened susceptibility to new intramammary infections (IMI), which account for over 50% of clinical mastitis (CM) cases occurring during the early postpartum weeks [1,2]. The risk of infection is particularly elevated during the initial stages of the dry period, prior to the complete formation of the keratin plug, a key physiological barrier that prevents pathogen entry [3]. Although this risk declines as involution advances it subsequently increases during the periparturient period [4]. The incidence of CM peaks within the first two weeks postpartum, exceeding the rates observed at any other stage of lactation [5].

To mitigate the risk of IMI during this vulnerable period, Blanket Dry Cow Therapy (BDCT) was introduced in the late 1960s as a key component of the National Five-Step Mastitis Control Plan, developed by the National Institute for Dairy Research (NIRD) [6]. This approach involves the intramammary administration of antibiotics to all animals within the herd, regardless of their individual infection status, with the objective of treating existing infections and preventing the onset of new cases [7]. In the following years, the protocol was widely embraced by countries with advanced dairy industries, significantly shaping the approach to mastitis management. In the United States and Canada, the implementation of BDCT reached approximately 72% and 88%, respectively, of dairy farms [8,9]. This trend was also evident in Europe, where, between 2005 and 2010, antimicrobial therapy was administered during the dry period to approximately 90% of lactating cows in the Netherlands [10], and in Ireland, BDCT became a standardized practice, fully implemented by 2015 [11]. As a consequence of this widespread adoption, significant modifications have been observed in the etiology of mastitis, including a marked reduction in the prevalence of contagious pathogens and a concurrent decrease in somatic cell count. For instance, in Canada, where BDCT was introduced in the late 1960s, *Streptococcus agalactiae* was nearly eradicated, and by 2005, the provincial average of Bulk Tank Somatic Cell Count (BTSCC) had decreased to 225,000 cells/mL [12]. Furthermore, according to a meta-analysis, the application of BDCT has been associated with a 39% reduction in the incidence of new IMI and a 78% increase in cure rates compared to the absence of this intervention [13].

Despite its benefits, BDCT has also raised major concerns due to its contribution to high antibiotic usage. Although the dairy sector uses relatively fewer antimicrobials compared to other livestock industries, such as swine production, it is well established that udder-related diseases are the primary drivers of antimicrobial use in this sector [14]. For example, in 2009, intramammary treatments accounted for over 60% of the total antimicrobial usage in Dutch dairy farms, with two-thirds of that use attributed to BDCT [15]. In the United States, this practice resulted in the administration of approximately 11 tons of antibiotics annually [16].

This extensive use of antibiotics has potential consequences, including the emergence of antibiotic-resistant microorganisms within both animal and, subsequently, human microbiota, as well as an increased risk of antibiotic residues in meat and dairy products [4]. As a result, growing concerns about the role of routine antibiotic use at dry-off in fostering antimicrobial resistance have increasingly called into question the sustainability of BDCT as a preventive strategy [17].

Antimicrobial resistance (AMR), widely recognized as a critical global threat to public health and the economy, is projected by the World Health Organization (WHO) to cause up to 10 million deaths annually and incur economic losses of approximately USD 100 trillion by the year 2050 [16]. Already in 2016, it was estimated that drug-resistant infections were responsible for the deaths of around 700,000 individuals each year. The severity of AMR lies in its capacity to compromise the effectiveness of antibiotics, which are essential for a wide range of medical interventions, including surgeries, chemotherapy, and other critical treatments. AMR disproportionately affects low- and middle-income countries and is driven by both the overuse of antimicrobials in human and veterinary medicine and inadequate access to effective treatments where they are most needed [18]. Thus, combating this crisis requires globally coordinated efforts, the reinforcement of antimicrobial stewardship programs, and significant investments in the development of new antimicrobial agents. Given these concerns and shifts in mastitis epidemiology, reassessment of dry cow therapy has become imperative [4,19]. In response, the EU aims to reduce antimicrobial use in livestock by 50% by 2030 [20], aligned with WHO recommendations. Accordingly, EU Regulation 2019/6, in force since January 2022, prohibits routine use of antimicrobials and imposes strict limits on prophylactic or metaphylactic intramammary use [21].

Consequently, an alternative to BDCT, extensively documented in the scientific literature and implemented in numerous countries, is the targeted administration of antimicrobial agents solely to cows identified as infected or at elevated risk of intramammary infection at the time of dry-off. This approach, known as Selective Dry Cow Therapy (SDCT), is particularly suited for herds with a low prevalence of contagious mastitis and consistently low BTSCC [22].

Numerous studies have evaluated the efficacy of SDCT, employing various cow selection methods, and examining their impact on udder health, milk production, and farm economics. The results derived from these studies are heterogeneous, and comparing them presents a challenge, as multiple factors can influence the effectiveness of SDCT, which vary significantly depending on the geographical context and the management conditions of each farm (e.g., dry period management practices, climatic conditions, pathogen distribution, and the use of internal teat sealants) [23]. Despite these variables, the majority of recent studies incorporating internal sealants in treatment protocols have concluded that SDCT is an effective and viable alternative to conventional antibiotic treatments, without adversely affecting udder health or farm economic performance. The objective of this review is to critically analyze and synthesize the existing literature on SDCT, with a particular emphasis on evaluating the cow selection methods used in the implementation of this practice, from the perspectives of efficacy and economic viability. Additionally, an analysis of global trends in SDCT adoption will provide a comprehensive understanding of the impact of this practice on animal health, economic outcomes, and the risks associated with AMR, an increasingly pressing global issue. A detailed assessment of the efficacy and safety of SDCT is essential to support its large-scale adoption in dairy farms, and a systematic, evidence-based approach will guide the decision-making process for those intending to implement SDCT.

## 2. Study Eligibility Criteria

This review included only primary research studies, published in English and peer-reviewed, which were found through searches in the Web of Science—Core Collection database. Most of the studies were conducted under natural exposure to disease, but experimental “challenge” trials and observational studies were also considered, as long as they met the eligibility criteria. To be included, studies had to focus on dairy cows after their first lactation and compare two treatment approaches used at dry-off: Blanket Therapy, where all udder quarters are treated with antibiotics, and Selective Therapy, where only infected cows or udder quarters are treated. Infection status was determined through bacterial culture, SCC, or a similar indicator. Studies were eligible if they reported at least one of the following outcomes: the frequency of intramammary infections (IMIs) at calving, IMIs within the first 30 days of the next lactation, cases of clinical mastitis during that same period, changes in SCC after treatment, milk production after treatment, antibiotic usage, or an economic evaluation of the treatment strategy.

The selected studies came from farms with different management practices, which may have affected the results. In many cases, the criteria for herd selection were not clearly described. Where mentioned, these criteria varied—for example, Cameron et al. [12] only included herds with low BTSCC (below 250,000 cells/mL), while other studies [24,25] included herds with a wide range of BTSCC levels, either by design or due to their sampling method. This variation in herd characteristics may affect how cows respond to Blanket versus SDCT, possibly explaining differences in the reported effectiveness of these treatments.

## 3. International Adoption of SDCT

After the introduction of SDCT as an alternative to BDCT, its implementation has gradually expanded across various countries, reflecting a progressive shift towards more targeted and rational use of antimicrobials. The Nordic countries have adopted SDCT since the 1970s, serving as an early model for the rational use of antimicrobials in veterinary medicine. A notable example is Norway, where this method was officially implemented in 2005. The results were evident: by 2009, the use of antibiotics during the dry period had decreased drastically, with only an estimated 0.05% of cows receiving treatment [26]. Another success story comes from the Netherlands, where the ban on BDCT in 2012 prompted the widespread adoption of SDCT. In 2013, the Royal Dutch Veterinary Association introduced a set of guidelines with clear criteria for applying this therapy [27], and the results were significant: by the end of that year, approximately 75% of farmers had adopted this strategy, although 80% of cows were still receiving antibiotic treatment. However, by 2017, 99% of farmers were following SDCT protocols, and the proportion of treated cows had dropped to 40% [28]. Crucially, studies reported no negative effects on udder health or milk production [27,29], confirming SDCT as a safe and effective alternative to BDCT. Similar success was seen in Norway, where reduced antibiotic use did not compromise mammary health: the average SCC was 125,000 cells/mL, and CM treatments fell by 73% from 1994 to 2018, with an additional 4.2% decrease between 2017 and 2018. Additionally, studies from Norway, Finland, Denmark, and Sweden report low levels of antimicrobial resistance in cattle, largely attributed to the prudent use of antibiotics in these countries [30]. A recent significant development occurred in Italy, where SDCT became mandatory as of 28 January 2022. This decision reflects an increasing European trend to regulate the use of antibiotics in the livestock sector and align local practices with international recommendations on reducing antimicrobial resistance. However, while the implementation of SDCT is advanced in some European countries, its global adoption remains limited. In the United States, for instance, only 10% of farms utilize this method, despite its potential to improve antibiotic management [31]. This reluctance can be partially explained by the perceived risks to cow health, stemming from negative experiences with early SDCT programs, which indicated an increase in CM incidence and SCC during early lactation [4,32,33]. Recent research, however, incorporating the use of internal teat sealants (ITSs), suggests that these risks may be mitigated, with minimal impact on udder health immediately after calving [19,22].

These findings underscore the importance of well-structured SDCT protocols, supported by accurate cow selection methods and clear institutional policies, to ensure both safety and long-term economic sustainability.

## 4. Selection Protocols

The decision to implement SDCT involves two levels of analysis: first, an evaluation at the herd level to determine whether this strategy is appropriate for the specific context of the herd, considering factors such as the prevalence of IMI, milk production, BTSCC and the incidence rate of CM, followed by an individual decision regarding the treatment of each cow [34].

### 4.1. Cow-Level Versus Quarter-Level Selection

During the dry-off period, three main strategies are used for administering antibiotics. The first is BDCT, where all cows receive antibiotics regardless of their infection status. Another approach is SDCT, in which only cows with at least one infected quarter are treated, and all four quarters of those cows receive antibiotics. The most targeted method is selective quarter-level therapy, where only the quarters confirmed to be infected are treated. These strategies aim to control mastitis effectively while reducing unnecessary antibiotic use [35,36].

The use of long-acting intramammary antibiotics as prophylactic treatment at dry-off is commonly recommended for all quarters. Evaluating its effectiveness at the quarter level assumes that the risk of infection in one quarter is independent from the others [37]. However, understanding inter-quarter dependency is essential to guide treatment strategies. If quarters act independently, selective quarter-level therapy is appropriate [38]. In contrast, interdependence would favor whole-cow treatment for optimal efficacy. Older studies that did not incorporate protective measures for untreated quarters, such as Internal Teat Sealant (ITS), frequently reported high levels of interdependence, with significantly increased incidence of new IMIs in previously uninfected quarters. Consequently, treating all quarters in cows with at least one infected quarter was recommended [24,39,40]. Berry et al. [37] also support this approach, noting that quarter interdependence persists during the dry period and cow-level susceptibility may influence infection risk in untreated quarters. Although the use of ITS was proposed as a risk-reduction strategy, limitations in sample size restricted firm conclusions regarding their protective benefit. Robert et al. [38] also examined IMI dynamics during the dry period, demonstrating that quarter interdependence continued through to calving, and that the chosen treatment strategy influenced infection risk. This conclusion aligns with Browning et al. [2], who found quarter-level therapy was associated with higher IMI rates compared to cow-level selective therapy and BDCT. It is worth noting that *Staphylococcus aureus* was the predominant causative agent at the time. However, pathogen prevalence has shifted significantly since then. Contagious mastitis agents have declined, and quarter interdependence is less evident with environmental pathogens, which pose similar infection risks to all quarters [41]. Thus, more recent studies conducted in well managed herds with low SCC, a lower prevalence of contagious pathogens, and utilizing ITSs have shown that interdependence may be mitigated and SDCT can be effectively implemented at either the quarter or cow level [36,42,43]. Therefore, whole-cow treatment is primarily warranted in herds where contagious pathogens have a high prevalence and represent the dominant cause of IMIs.

### 4.2. Methods Applied for Cow Selection

According to the existing literature, the most significant challenge in the successful application of SDCT lies in the methodologies used for cow selection [44]. Therefore, to ensure an effective selection for treatment, it is imperative to utilize methods that are highly accurate, straightforward to apply and interpret, economically feasible, and accessible. It is also crucial that the detection of infected or infection-susceptible animals is conducted with high sensitivity and specificity [34]. Such methods enable the precise identification of cows or quarters that require antimicrobial treatment, while also distinguishing those that can safely be left untreated [43]. A low diagnostic sensitivity may lead to false-negative results, whereby infected cows remain untreated, while reduced specificity increases the likelihood of false positives, potentially resulting in unwarranted antimicrobial administration and contributing to overuse [34]. Additionally, selection thresholds should be adapted based on the health status of each herd to optimize management strategies and the responsible use of antimicrobials [45].

Among the procedures used for IMI identification are: SCC (measures the number of somatic cells in milk, indicating inflammation or infection), pathogen-specific techniques (bacteriological cultures, PCR) (detect and identify specific bacterial pathogens causing infection), California Mastitis Test (CMT) (a cow-side test that estimates somatic cell levels through a color change reaction), Differential Somatic Cell Count (DSCC) (distinguishes between types of immune cells to improve infection diagnosis), Electrical Conductivity testing (measures changes in milk conductivity caused by infection-related alterations), Lactate Dehydrogenase (an enzyme elevated during tissue damage and infection), and N-acetyl-β-d-glucosaminidase (an enzyme marker associated with mastitis) [46].

#### 4.2.1. Bacteriological Culture

Culture-based SDCT methods aim to provide an accurate etiological diagnosis of IMIs, enabling the precise identification of animals requiring antimicrobial intervention and ensuring targeted treatment against the specific bacterial pathogen involved [46]. Despite its recognized diagnostic accuracy, the implementation of culture-based SDCT methods is often hindered by practical limitations such as restricted access to microbiological laboratories, the labor-intensive nature of the process, and the relatively high costs associated with standard bacterial culture protocols [47]. These challenges may significantly limit the feasibility of widespread adoption, especially in small- to medium-scale dairy farms. Another frequently discussed drawback lies in the occurrence of CM cases with negative bacteriological findings—an issue that can affect up to 30% of the tested samples, depending on the culture system employed. This high rate of false-negative results is thought to stem from several factors, such as a low number of viable bacterial cells below the detection threshold, intermittent shedding of pathogens such as *Staphylococcus aureus*, or delayed sampling, by which time the host’s immune response may have already cleared the infection [20]. These diagnostic blind spots raise concerns regarding the under-detection of IMIs and call into question the reliability of single-sample testing. As a potential solution, several studies advocate for the interpretation of consecutive milk samples to minimize both false-negative and false-positive results—the latter often caused by contamination at the teat end [48,49]. For instance, Buelow et al. [50] reported a sensitivity of 91% in detecting *S. aureus* from a single milk sample, compared to a gold standard involving two positive samples collected over a six-day interval. The same study suggested that using consecutive sampling could improve IMI detection rates at dry-off by an additional 7%.

In an effort to overcome the logistical and financial constraints of laboratory-based bacterial cultures, on-farm diagnostic methods have been investigated as more practical alternatives. Cameron et al. [12,22,51] evaluated the effectiveness of the Petrifilm culture system, a user-friendly tool that delivers results within 24 h and can be interpreted directly by farm personnel. The system demonstrated a sensitivity of 85.2% and a specificity of 73.2% in detecting IMIs in cows with low SCC at dry-off [51], suggesting its potential as a reliable diagnostic aid in SDCT protocols. Further studies conducted by the same group assessed the broader impact of implementing Petrifilm-based SDCT programs on udder health, post-calving IMI incidence, milk production, and SCC. The findings indicated that selective treatment using this method was comparably effective to BDCT, managing to maintain udder health and milk quality while significantly reducing antimicrobial usage [12,22]. Supporting these outcomes, Kabera et al. [35] reported even greater reductions in unnecessary antibiotic use when applying the Petrifilm-based protocol at the quarter level rather than the whole-cow level, further reinforcing the benefits of a more granular diagnostic approach.

However, despite its diagnostic merit, culture-based testing—whether laboratory or on-farm—still faces limitations. Sampling and testing all cows remain labor-intensive and economically burdensome, which restricts the widespread adoption of such protocols across the dairy sector [19,52]. As such, the challenge lies not only in the diagnostic accuracy but also in the feasibility of integrating these tools into routine farm workflows.

#### 4.2.2. Somatic Cell Count

Considering the limitations associated with bacteriological analysis, numerous studies have investigated more practical and economically viable alternatives, reporting promising results based on the evaluation of inflammatory markers [53]. Globally, the primary inflammatory marker employed as a monitoring tool is the somatic cell count (SCC) [54,55,56,57]. One of the main advantages of SCC-guided SDCT lies in the efficient utilization of data already available on farms. Most modern, large-scale dairy operations are integrated into routine testing programs and rely on computerized management systems, which helps reduce both costs and implementation delays [31,42]. Moreover, since this approach uses composite milk samples collected from all four quarters [2], treatment decisions are made at the cow level. This simplifies and enhances implementation by farm personnel compared to protocols that require quarter-level treatment [31].

SCC thresholds are widely used to assess the infection status of animals, with values above the threshold suggesting potential IMIs, while values below it generally indicate a healthy status [58]. Internationally, the threshold of 200,000 cells/mL is widely accepted and plays a significant role in epidemiological monitoring, providing a balance between sensitivity and specificity and substantially minimizing diagnostic errors [54,56,58,59,60]. Nevertheless, signs of udder inflammation have occasionally been detected even at SCC values below 100,000 cells/mL [61,62,63], with some contagious pathogens inducing SCC levels below this threshold [64]. For instance, Djabri et al. [65] demonstrated that *Staphylococcus aureus* may elicit only a moderate increase in SCC, which could result in misclassification of infected cows when SCC is used as the sole indicator of subclinical mastitis at dry-off. In this context, numerous researchers have investigated various SCC thresholds, considering either single or multiple monthly test results, in an effort to optimize the sensitivity and specificity of identifying infected animals (Table 1). Additionally, it has been shown that incorporating the history of CM into SCC-based decision-making algorithms slightly enhances their sensitivity in identifying infected cows [15,19,31,36,44,52].

Thus, Østerås et al. [67] found that the geometric mean SCC from the last three months of lactation was more strongly associated with persistent major pathogen IMIs in the subsequent lactation than SCC from a single monthly test or from the entire lactation. Torres et al. [44] support this conclusion, adding that the most effective strategy is the application of a threshold of 200,000 cells/mL for cows without CM episodes during lactation and a threshold of 100,000 cells/mL for those that experienced CM in the first 90 days of lactation. The inclusion of an additional requirement, specifically setting a lower SCC threshold at the time of drying off for cows with a history of CM, resulted in the highest classification accuracy rate, with a sensitivity of 70% and specificity of 63%. Similar findings were reported when applying this approach across various datasets [68]. However, combining recent sequential SCC data with a test performed precisely at dry-off improves diagnostic precision, as demonstrated by Vasquez et al. [19], who achieved over 90% accuracy in identifying uninfected quarters using a 200,000 cells/mL threshold. Supporting this, another study found that relying solely on the final SCC value prior to dry-off yielded a sensitivity of just 40%, whereas performing the test exactly at dry-off increased sensitivity to 64% [66]. Building on these findings, several recent studies have highlighted that a single SCC test performed at dry-off yields results comparable to those obtained from the last three monthly recordings [52]. This approach has proven to be both effective and practical, offering good diagnostic accuracy, a high negative predictive value (NPV), and a significant reduction in antimicrobial use at dry-off [42]. One of the most compelling examples of successful implementation comes from the Netherlands, where antimicrobial treatment during the dry period is allowed only when a clear indication of IMI is present. This criterion, typically based on the SCC value from the last milk sample before dry-off, has been part of SDCT protocols since 2013, without adverse effects on udder health [27].

Although some authors identified 100,000 cells/mL as the optimal SCC threshold due to its balanced sensitivity and specificity (Table 1) [52,66], subsequent studies have shown that effective outcomes can also be achieved using the internationally accepted cutoff of 200,000 cells/mL. For example, Kabera et al. [42] advocated for a simplified strategy that applied this higher threshold uniformly across all cows, based on findings indicating minimal differences in predictive performance compared to the lower cutoff. This approach was further supported by evidence demonstrating that a single SCC test conducted at dry-off, combined with CM history and the 200,000 cells/mL threshold, significantly reduced antimicrobial usage without compromising udder health [69]. An important consideration is that SCC is not a fully accurate indicator, as it can be influenced by factors such as milk yield, lactation stage, breed, and herd-specific variables like infection prevalence. Consequently, defining SCC thresholds to distinguish between infected and uninfected cows may require adjustments based on age group, breed, or herd-specific characteristics [34]. Given the imperfect nature of diagnostic tests, optimizing the selection process may involve combining multiple criteria to enhance NPV. One feasible approach is to introduce a second diagnostic test for cows initially classified as uninfected based on SCC. However, the utility of additional testing, such as bacterial culture, for animals already identified as infected based on elevated SCC remains debatable, as the unnecessary treatment of uninfected cows has limited consequences at the herd level, whereas false negatives may lead to more serious outcomes [52].

Therefore, the convergence of data presented in the literature indicates that, although approaches based on multiple sequential tests may provide a higher predictive value, the practical applicability and efficiency of a simplified algorithm, centered on the final SCC measurement combined with clinical history, are increasingly supported in the current context of antimicrobial reduction.

#### 4.2.3. Differential Somatic Cell Count

Another criterion for identifying cows that require antimicrobial treatment under SDCT protocols is the differential analysis of the primary somatic cell types found in milk [21]. While traditional SCC offers a general indication of mammary health by quantifying the total number of cells present, it does not distinguish between the types of immune cells involved. In contrast, the differential somatic cell count (DSCC) allows for the identification of the relative proportions of lymphocytes, macrophages, and polymorphonuclear leukocytes (PMNs), immune cell types that play distinct roles in mammary gland defense [70]. The introduction of DSCC, as described by Damm et al. [71], has brought a valuable layer of specificity to milk quality monitoring. Measured using high-throughput flow cytometers, DSCC represents the combined percentage of PMNs and lymphocytes out of the total SCC. Notably, DSCC values tend to rise in tandem with SCC, particularly in the presence of inflammation, and elevated DSCC percentages are increasingly associated with IMIs [71]. While SCC alone may reflect a response to various physiological conditions, the increase in DSCC—more directly linked to the activation of immune cells—can offer a more pathogen-specific signal [72,73].

**Figure 1:**
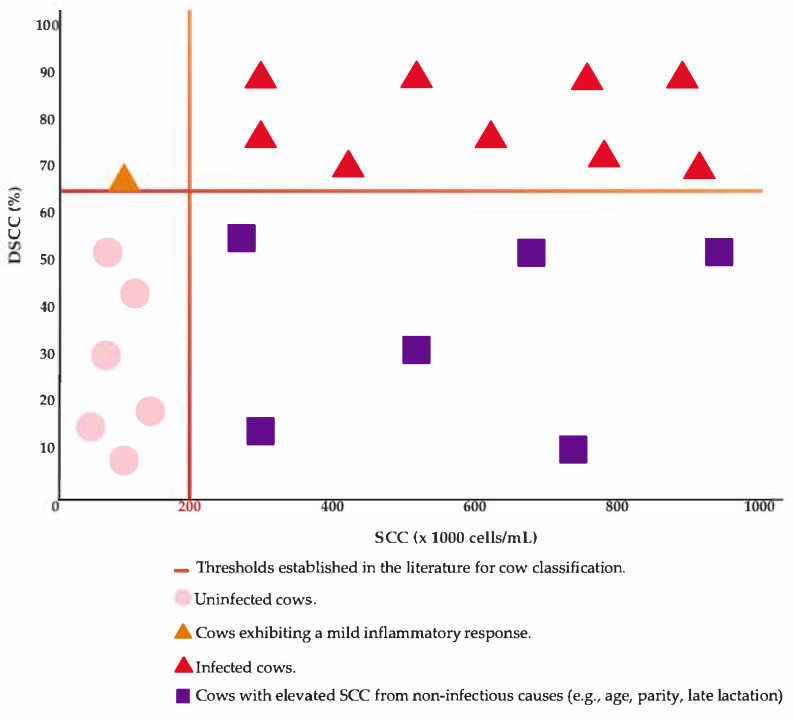
Cow classification for SDCT based on combined DSCC and SCC values. Thresholds for infection were set at 200,000 cells/mL for SCC and 65% for DSCC. Light pink circles represent uninfected cows; the orange triangle indicates a cow with a mild inflammatory response (high DSCC, low SCC); red triangles denote infected cows (high SCC and DSCC); purple squares represent cows with elevated SCC due to non-infectious causes such as age, parity, or late lactation.

What makes DSCC particularly promising is its ability to enhance diagnostic sensitivity and specificity when used in conjunction with SCC. For instance, in the study conducted by Schwarz et al. [74], SCC alone, at thresholds of 100,000 and 200,000 cells/mL, identified 65.63% and 46.88%, respectively, of cows infected with major pathogens. Similarly, DSCC alone, evaluated at thresholds of 50%, 60%, and 70%, yielded comparable or slightly improved detection rates. However, the true diagnostic advantage became evident when SCC and DSCC were combined: the sensitivity increased to 78.13% when using SCC > 100,000 cells/mL and DSCC > 50%. Even when applying the higher SCC threshold of 200,000 cells/mL, the addition of DSCC still led to a substantial improvement in detection. These findings suggest that, while each parameter has limitations when used in isolation, their combined application significantly enhances diagnostic accuracy. In this context, farms that rely solely on SCC for dry-off treatment decisions could benefit from incorporating DSCC into their protocols, thereby increasing the likelihood of detecting subclinical infections that may remain unnoticed when using traditional SCC thresholds alone (Figure 1).

Another important aspect supporting the notion that DSCC should not be interpreted in isolation is its dynamic nature, which is influenced by factors such as parity and lactation stage, though not necessarily following the same pattern as SCC. DSCC has shown variable trajectories throughout lactation: some studies report increases with advancing days in milk (DIM) [74], while others observed a decline [75]. Dal Prà et al. [76] explored this complexity by analyzing DSCC in healthy and subclinically infected quarters across different SCC classes. In quarters with SCC between 50,000 and 200,000 cells/mL, generally considered uninfected, DSCC rose from 44% to 55% overall, but in some cases, it reached up to 70%, particularly around calving or peak lactation. In infected quarters (>200,000 cells/mL), DSCC increased to 65% or higher, again peaking during physiologically stressful periods. Even so, further research in diverse herds is necessary to validate these findings and better understand the factors influencing DSCC dynamics under varying management and environmental conditions.

Equally important for SDCT implementation is the need to individualize DSCC thresholds. Studies to date have proposed cut-offs between 65% and 72% for detecting IMIs, but the optimal value likely varies depending on farm conditions, pathogen prevalence, and cow demographics [71].

Lastly, considering that SCC thresholds of >200,000 cells/mL remain the clinical standard for identifying IMIs, and that many subclinical cases remain undetected at this level, the inclusion of DSCC as a complementary tool represents an important step forward [76]. Its integration into SDCT protocols could not only improve the identification of at-risk cows but also reduce unnecessary antimicrobial use, aligning with broader goals of sustainable herd health and antimicrobial stewardship.

#### 4.2.4. California Mastitis Test

The efficacy of the California Mastitis Test (CMT) as a selection criterion for SDCT has also been extensively investigated. The early implementation of a CMT-based protocol by Poutrel and Rainard [77] led to the correct identification of approximately 80% of infections caused by major pathogens. However, only 23% of infections caused by minor pathogens were detected, and 13% of uninfected quarters were incorrectly classified as positive. These results highlighted the diagnostic value of CMT, while also emphasizing its limitations, particularly in its reduced ability to detect minor pathogen infections. Recent studies have further supported these findings, demonstrating that CMT is more reliable when minor pathogen infections are excluded from the analysis [41,78]. For example, Sanford et al. [55] reported a sensitivity of 70% and a specificity of 48%, noting that its applicability is greater in herds with an IMI prevalence below 15%. Another limitation of this practice is that the sensitivity of CMT for detecting IMIs is significantly reduced when the test is performed by farmers or untrained personnel [41]. The subjectivity of the test presents a considerable drawback, as accurate interpretation heavily relies on the expertise of qualified personnel, making its effectiveness dependent on proper training and experience.

## 5. Teat Sealants

Although the literature widely supports the use of SDCT, a major concern remains the vulnerability of untreated quarters to IMIs during the dry period. This vulnerability is due to the delayed formation of the keratin plug—a key physiological defense that blocks the teat canal and acts as a physical barrier against bacterial invasion [7]. Data from Williamson et al. [40], based on 657 quarters, based on 657 quarters, showed that only 50% of quarters closed within 7 days after dry-off, with 5% remaining open beyond 90 days. Similarly, 83% of CM cases occurred within the first 21 days, highlighting early susceptibility. As an alternative to antibiotics in healthy cows, ITSs provide an effective preventive approach. Typically made of inert substances like bismuth subnitrate, ITSs mimic natural keratin plugs by creating a lasting physical barrier in the teat cistern until removed at the first milking postpartum [79]. Their effectiveness is well documented in comparative studies [4,55,80], showing similar [27,35,81,82] or even greater effectiveness than antimicrobials alone [83] in preventing new IMIs, dry period mastitis [80], early lactation infection [12,36], and preserving milk quality [22] (Table 2). ITSs are also recommended combined with antibiotics for infected cows or herds with high SCC, where the risk of new IMI remains elevated [84]. Biggs [23] reported that adding ITSs to antibiotic therapy resulted in higher cure rates during the dry period compared to antibiotics alone. Without ITSs, some cows infected at dry-off initially achieved bacteriological cure but became reinfected before calving, leading to perceived treatment failure. Using ITSs together with antibiotics helped maintain these early cures by lowering the risk of reinfection. Nonetheless, in cows with low SCC, several studies [12,19,35,80,84] found no additional benefit from combining ITSs with antibiotics. The importance of ITSs is further highlighted in studies where sealants were not used, leaving untreated cows vulnerable to infection throughout the dry period. In these cases, SDCT was associated with a higher risk of new IMIs [1], increased incidence of CM [33], elevated SCC [33,68], and reduced milk yield in the subsequent lactation [68], compared to BDCT (Table 2).

An important consideration is that the efficacy of TSs varies among herds, depending on infection prevalence, dominant pathogens, mastitis control practices [11], and the accuracy of IMI diagnosis [80]. Notable examples include the findings of McParland et al. [85] and Clabby et al. [11], which indicate that low-SCC cows (<200,000 cells/mL) treated solely with ITSs at dry-off exhibited significantly higher SCC levels in the subsequent lactation compared to those receiving combined therapy (ITSs and antibiotics) (Table 2). These discrepancies from other studies are most likely attributable to regional differences in pathogen profiles—specifically, the predominance of *Staphylococcus aureus* observed in the herds analyzed in these studies.

Another type of sealant occasionally employed in dry-cow management is the external TSs, which, though less commonly used, forms a protective film on the teat surface and is typically applied via post-dip cups. Despite practical limitations, such as difficult application, limited persistence, and the need for repeated reapplication [86], these products have shown encouraging outcomes in the context of SDCT. For instance, Vasquez et al. [19] reported no significant differences between external TS and BDCT in terms of linear SCC, risk of new IMI, milk production, culling rates, or CM incidence (Table 2).

**Table 2:** Summary of studies comparing blanket and selective dry cow therapy based on different selection methods and the presence or absence of internal teat sealants.

Bibliographic References	Method	Internal Teat Sealants	Results
Rindsig et al. [1]	Infusion in all quarters only if: History of 1 CM during the current lactation. A score of +2 or +3 on the 2 CMT (in any quarter on the day of drying-off). 3 BTSCC > 500,000 cells/mL.	no	Similar cure rate of existing infections: BDCT—85.4%; SDCT—88.2%. Slightly higher new IMI rate during the dry period in SDCT (6.5%) compared to BDCT (3.1%). Higher incidence of CM after calving in SDCT (7.8%) than in BDCT (4.6%).
Browning et al. [24]	BTSCC: 100–400,000 cells/mL. Quarters were considered infected if 2 out of 3 consecutive bacteriological samples contained the same pathogen. Uninfected cows: randomly assigned to receive either treatment in all quarters or no treatment. Infected cows: allocated to receive treatment either in all quarters or only in the affected quarter.	no	Significantly higher new IMI rate during the dry period in cows treated only in infected quarters (15.3%) compared to those treated in all quarters (4.3%). *Streptococcus uberis* was more frequently isolated in the quarter-treated group, both at calving and mid-lactation. No significant differences in the incidence of CM, although group-level variations were observed in early lactation.
Browning et al. [2]	Refer to Browning et al. [24]	no	Cows treated selectively at the quarter level had a higher percentage of infected quarters at calving (10.0%) compared to those treated selectively at the cow level (7.9%) or those treated universally (6.8%), though no significant differences were observed between strategies. By mid-lactation, the prevalence of infection was similar across all treatment strategies.
Østerås & Sandvik [87]	Cows with >100,000 cells/mL in the last 2 tests and a positive CMT or a major pathogen identified in 1+ quarters. Randomized study: Group A = Control. Group B = Placebo. Group C = Long-acting antibiotic in infected quarters. Group D = Short-acting antibiotic administered every two days for 8 days in infected quarters. Treatment at the quarter level (except if >3 quarters were infected).	no	No effects on the culling rate. Cows in the control group exhibited a higher incidence of clinical mastitis, elevated SCC, and a lower average milk yield per lactation.
Berry & Hillerton [4]	Quarter-level AB administration when: the same pathogen was isolated in two consecutive samples, in two out of three samples, or in a single sample with SCC > 200,000 cells/mL and at least twice as high as in the other quarters.	no	Untreated groups showed a higher incidence of new IMIs. Treatment was effective in eliminating infections caused by *Streptococcus uberis* and partially effective against *Staphylococcus aureus*. The absence of treatment increased the risk of coinfections (*S. uberis* and coliform infections).
Bradley et al. [82]	BTSCC < 250,000 cells/mL. Uninfected cows: SCC < 200,000 cells/mL in the last three monthly tests and no CM during this period—only ITS or AB + ITS (at the quarter level). Infected cows: all other animals—only ITS or ITS + AB (at the quarter level).	yes	Infected cows: The combined treatment ITS + AB increased the chances of pathogen elimination and reduced the risk of CM in the first 100 days of lactation compared to AB alone. Uninfected cows with low SCC: The combination of ITS + AB did not significantly reduce the risk of infection compared to ITS alone, but was associated with a higher prevalence of IMI with coagulase-positive staphylococci and Streptococcus spp.
Rajala-Schultz et al. [68]	Low risk: SCC < 200,000 cells/mL in the last 3 monthly tests and no CM during this period; CM in the first 90 days of the previous lactation but SCC < 100,000 cells/mL during the rest of lactation randomly assigned to receive treatment or not. High risk: All other animals—all cows were treated.	no	The MY of cows with low SCC, whether treated or untreated, did not differ significantly in the following lactation. Treated cows with low SCC had a 16% lower SCC (35,000 cells/mL), but the effect varied between farms.
Scherpenzeel et al. [33]	SCC < 150,000 cells/mL for primiparous cows. SCC < 250,000 cells/mL for multiparous cows. No CM present. Quarter-level analysis (split by quarters).	no	The incidence of CM was 1.7 times higher in untreated quarters, especially during the first 21 days after calving. Significantly higher SCC was observed in untreated quarters at calving and during the first 14 days postpartum.
Cameron et al. [12]	BTSCC < 250,000 cells/mL. On-farm Petrifilm culture for cows with SCC < 200,000 cells/mL in the last three tests and no CM during this period. Negative culture: SDCT –ITS only. Positive culture: BDCT—AB + ITS.	yes	No differences were observed between the BDCT and SDCT groups in terms of bacteriological cure (per quarter), new IMIs (during the dry period), IMIs at calving, or CM (during the first 120 days of lactation).
Cameron et al. [22]	Refer to Cameron et al. [12]	yes	Similar MY between groups (BDCT: 39.3 kg, SDCT: 39.0 kg). The natural logarithm of SCC was also similar (BDCT: 3.95 vs. SDCT: 3.97).
Tho Seeth et al. [25]	Group A: On-farm Petrifilm culture. (+) Positive: AB + ITS. (–) Negative: ITS only. Group S: SCC < 200,000 cells/mL at last test and no CM in the previous lactation –ITS only. SCC ≥ 200,000 cells/mL or CM in the previous lactation AB + ITS. Group C (Control, BDCT): AB + ITS.	yes	Group C achieved the best results in terms of udder health, especially bacteriological cure during the dry period. Groups S and A showed only marginally weaker results. A significant difference in bacteriological cure was observed between Group S and Group C. The risk of new IMIs was similar across all three groups.
Vasquez et al. [19]	Low risk: SCC ≤ 200,000 cells/mL at the last test; Average SCC over the last 3 monthly tests ≤ 200,000 cells/mL; No signs of CM at dry-off. A maximum of one CM episode during the current lactation—randomly assigned to receive BDCT or SDCT. High risk: Treated with BDCT.	external sealant	No significant differences between treatment groups in terms of risk of new IMI, milk production, linear scores, culling events, or CM. Slightly higher bacteriological cure rates in animals treated with AB.
McParland et al. [85]	Low risk: SCC < 200,000 cells/mL throughout the previous lactation and no CM—randomly assigned to BDCT + ITS or SDCT. High risk: BDCT + ITS.	yes	Cows treated only with ITSs produced, on average, 0.67 kg/day more milk, but had a slightly higher SCC throughout lactation compared to cows treated with ITS + AB. Weekly SCC did not differ between cows with SDCT and those in the high-risk group. Cows with ITSs had a 2.7- times higher risk of bacterial presence in early lactation, compared to those in the low-risk group treated with the combined approach, and 1.6 times higher compared to high-risk cows.
Rowe et al. [36]	Culture-SDCT: Treatment AB + ITS only for bacteriologically positive quarters. SCC-SDCT: Treatment with AB + ITS only for cows with SCC > 200,000 cells/mL in any monthly test during the current lactation and ≥2 cases of CM during the same lactation. BDCT: All quarters treated with Ab + ITS.	yes	The risk of bacteriological cure, new IMIs during the dry period, and new IMIs after calving were similar across the three groups.
Rowe et al. [31]	Refer to Rowe et al. [36]	yes	The risk of culling, CM in the first 120 days of lactation, SCC, and MY were similar across the treatment groups.
Kabera et al. [35]	Petrifilm used for on-farm culture SDCT: Positive culture: AB + ITS; Negative culture: ITS only. BDCT: AB for infected quarters, ITS for healthy quarters, AB + ITS for infected quarters, sealant only for healthy quarters.	yes	There were no significant differences between groups in terms of acquisition of new IMIs, persistence of existing IMIs, incidence of clinical mastitis in the following lactation, average SCC score, and milk production.
Zecconi et al. [63]	Antibiotic treatment was administered when: the SCC at the last test exceeded 100,000 cells/mL in primiparous cows and 200,000 cells/mL in multiparous cows.	yes	The use of an ITS resulted in a higher bacteriological cure rate and significantly reduced the incidence of new IMIs. The proportions of negative cows (49.1% vs. 49.3%), transient infections (24.8% vs. 27.3%), and persistent IMI (26.1% vs. 23.5%) were very similar at dry-off and after calving.
Clabby et al. [11]	BTSCC < 250,000 cells/mL. Low risk: SCC < 200,000 cells/mL in the previous lactation—treated with ITS or ITS + AB. High risk: all other cows—treated with ITS + AB.		The logarithmic value of SCC in cows from the low-risk group treated only with ITSs was significantly higher compared to cows from the group treated with ITS + AB but showed no significant differences compared to cows from the high-risk group in the following lactation. The response to treatment varied depending on the herd studied.
Goncalves et al. [16]	Healthy cows: no bacteria isolated, no CM, and SCC < 200,000 cells/mL in the last 3 monthly tests –ITSs only. Cows with subclinical mastitis: positive culture in one quarter and SCC > 200,000 cells/mL—AB + ITSs.	yes	The bacterial diversity was similar between healthy quarters and those that were cured, regardless of the dry-off protocol. Healthy cows treated only with ITS showed a higher abundance of beneficial or commensal bacteria in the mammary gland.
Lipkens et al. [88]	BTSCC < 250,000 cells/mL BDCT: cows with an odd ear tag number—treated with AB + ITSs. SDCT: cows with an even ear tag number—received AB only if the infection assessment algorithm indicated by Lipkens et al. [53] required it.	yes	The differences in SCC between the SDCT and BDCT groups were minimal, with slightly better values for the SDCT cows. Cows in the SDCT group had a higher average daily milk yield during the first 100 of lactation compared to those in the BDCT group.
Pavesi et al. [89]	No CM and SCC < 200,000 cells/mL throughout lactation—2 groups: cows treated with ITSs only; cows treated with ITSs + AB.	yes	SDCT did not negatively affect milk production or udder health, with SCC values being similar in both groups. No CM was observed in the first 100 days, even in the group treated with ITSs only. Milk microbiota remained stable.
Paiva et al. [69]	SDCT1: AB administered if SCC > 200,000 cells/mL at any of the monthly tests or if the cow had ≥2 cases of CM during the current lactation. SDCT2: AB administered if SCC > 200,000 cells/mL at the last test or if there was any case of CM during the current lactation. BDCT: control group.	no	SDCT did not affect the risk of new IMIs or the cure of existing ones. There were no significant differences between SDCT and BDCT in terms of CM, SCC, MY, or culling risk during the first 180 days of lactation.
D’Amico et al. [90]	3 groups. S-SDCT: Quarter-level treatment only if SCC ≥ 200,000 cells/mL. C-SDCT: Quarter-level treatment only if bacterial growth was detected. BDCT: Treatment applied to all quarters.	yes	Antimicrobial use: Cows in both SDCT groups received fewer antimicrobial treatments than those in the BDCT group; C-SDCT cows were treated less than S-SDCT cows. Linear score at first test: Higher in SDCT groups (BDCT: 1.8; S-SDCT: 2.2; C-SDCT: 2.2). Other outcomes: No significant differences between groups.

CM—Clinical Mastitis. CMT—California Mastitis Test. BTSCC—Bulk Tank Somatic Cell Count. SCC—Somatic Cell Count. AB—Antibiotic. ITS—Internal Teat Sealant. MY—Milk Yield.

## 6. The Impact of SDCT

The implementation of SDCT has emerged as a promising strategy for reducing antimicrobial usage, thereby contributing to the mitigation of antimicrobial resistance [73]. However, for this approach to gain broader acceptance among dairy farmers and to be considered a viable alternative to conventional BDCT, its efficacy must be at least equivalent in terms of clinical outcomes. This includes the absence of adverse effects on the incidence and resolution of IMIs during the dry period, as well as the preservation of udder health and subsequent lactational performance [35]. Recent studies underscore that, although herd-level variability remains a challenge, the adoption of SDCT can sustain long-term udder health and milk yield at levels comparable to traditional strategies [27,91]. Nevertheless, its adoption across Europe remains inconsistent, largely shaped by the perceived risk of increased IMI rates and the potential need for higher antibiotic use during the subsequent lactation [88].

### 6.1. New Intramammary Infections, SCC, and Clinical Mastitis Rate

Over time, research on the implementation of SDCT has contributed to an increasingly nuanced understanding of the potential impact this approach may have on key parameters used to evaluate udder health. Early studies, conducted in herds where contagious pathogens were predominant and where ITSs were not used, consistently reported a significantly lower incidence of new infections and CM, as well as lower SCC values in cows treated with BDCT compared to those treated selectively [1,2,4,32,33,92,93]. These findings initially reinforced skepticism toward SDCT during its early stages of investigation and application. However, as the selection criteria for eligible animals have become more refined, the etiology of mastitis has shifted—characterized by an increasing prevalence of minor pathogens—and ITSs have been incorporated into SDCT protocols, the conclusions drawn from recent studies have become more balanced. A notable example is the study by Cameron et al. [12], which not only found no significant difference in the incidence of new IMIs between BDCT and SDCT groups, but actually reported a higher incidence of fungal infections in cows receiving BDCT. This finding challenges the assumption that BDCT is inherently risk-free and highlights its potential unintended consequences. Furthermore, several recent studies focusing on the optimization of selection protocols suggest that, when these criteria are well-defined and rigorously applied, particularly in herds with low pathogen pressure, SDCT can provide a level of protection comparable to that offered by conventional BDCT [19,22,25,31,36,85,89]. Additionally, some authors have even reported lower SCC values in selectively treated groups [88]. Another compelling example supporting the effectiveness of SDCT comes from the Netherlands, where no significant changes in SCC or CM incidence were observed at the herd level following the nationwide implementation of SDCT between 2011 and 2015 [27]. Nevertheless, the generalizability of these results to other farming and management systems remains uncertain, particularly in countries such as the United States, Canada, or the United Kingdom, where BDCT continues to be widely applied [94].

### 6.2. Milk Yield

IMIs present at the time of parturition, either as a result of the persistence of pre-existing infections or due to the establishment of new infections during the dry period, have been associated with a reduction of approximately 5% in milk production during the subsequent lactation [95]. In this context, the control and prevention of postpartum IMIs represent key factors for maximizing lactational performance. Additionally, various authors emphasize that untreated quarters have a significantly higher risk of contracting new infections during the dry period, which requires the application of protective measures, particularly for cows that do not receive treatment [1,4].

The scientific literature shows that SDCT does not have a clear negative impact on milk production in the subsequent lactation when cows are correctly selected. For example, studies by Rajala-Schultz et al. [68] and S. L. Berry et al. [96] demonstrated that, in herds characterized by a low prevalence of contagious pathogens and low SCC, omitting antimicrobial therapy at dry-off did not significantly affect milk production, even in the absence of ITSs. Similar or even superior results have been reported in other studies where, by using various selection criteria and applying intramammary sealants, milk production in the subsequent lactation was found to be comparable between cows treated with SDCT and those treated with BDCT [19,22,31,35] (Table 2). Moreover, a study conducted in Ireland provides a strong argument in favor of SDCT: cows with low SCC (considered negative according to the selection algorithm) that were randomized to receive only ITSs recorded an increase in average daily milk production of +0.67 kg over the entire lactation, compared to cows randomized to receive both ITSs and antibiotic treatment [85]. These conclusions are also supported by Lipkens et al. [88], who reported that cows in the SDCT group had higher average milk production values during the first 100 days of lactation than cows in the BDCT group, suggesting that reducing antibiotic use does not compromise, but may even improve, performance in healthy cows. In contrast, in herds with higher risk factors, the importance of antibiotic administration was highlighted, with a significant increase in milk production (by 91 kg in the subsequent lactation) observed in cows in the BDCT group [17].

### 6.3. Antibiotic Consumption

The implementation of SDCT is a critical component in efforts to reduce antibiotic use in the dairy production sector, a particularly relevant objective in the context of global public health concerns and food safety regulations. Although antimicrobials remain an essential tool for the prevention and control of mastitis during the dry period, providing high cure rates for infections caused by susceptible pathogens [13], the emergence of new IMIs cannot be entirely prevented. This is particularly true in cases where pathogens exhibit resistance or in the later stages of the dry period, when antibiotic concentrations may fall below the minimum inhibitory concentration (MIC) threshold [97]. In a study conducted by Browning et al. [2], inefficient antimicrobial use within the BDCT approach was highlighted, revealing that approximately 1400 tubes of antibiotic were administered to prevent just 25 new infections during the dry period. Furthermore, the BDCT strategy required twice as many antimicrobial resources to eliminate an existing infection compared to SDCT. These findings are further supported by more recent data, which indicate that over 85% of quarters did not exhibit bacterial isolation or elevated SCC, demonstrating that, in the absence of SDCT, unnecessary and unjustified antibiotic use would have occurred [16].

The literature consistently supports the notion that the application of SDCT significantly reduces antimicrobial consumption, often without negatively impacting udder health or subsequent lactational performance. For instance, Cameron et al. [12] evaluated an SDCT protocol based on historical SCC and the use of on-farm rapid Petrifilm culture (Table 2), achieving an overall 21% reduction in antibiotic use during the dry period. While the application of this protocol at the cow level led to moderate reductions in antibiotic use across the studied herds, the authors suggest that implementation at the quarter level may further reduce usage at the end of lactation. In a similar direction, Kabera et al. [35] confirmed the efficacy of a comparable protocol, achieving a 58% reduction in antibiotic use. Additional studies from North America and Europe align with these findings: McParland et al. [85] reported a 48% reduction, and Vasquez et al. [19] 60%, Lipkens et al. [88] 22%, and Scherpenzeel et al. [33] a remarkable 85% reduction. Rowe et al. [36] also demonstrated that SDCT can effectively reduce antibiotic use during the dry period, though considerable variability was observed among farms, with wide ranges in both culture-based SDCT (32–68%) and algorithm-based SDCT (19–68%) approaches. This variability is likely influenced by factors such as the prevalence of intramammary infections, incidence of CM, and the risks of contamination during milk sample collection for rapid culture testing.

At the national level, the Netherlands provides a compelling case where mandatory SDCT implementation has resulted in a significant 36% reduction in antimicrobial use [28], highlighting the substantial impact of these strategies in reducing antibiotic use on a broader scale. Adopting SDCT, based on thorough quarter-level health assessments and the accurate identification of cows needing treatment, can substantially reduce antimicrobial use in dairy herds while maintaining animal health and productivity in the following lactation.

### 6.4. Economy

Farmer decisions regarding dry period therapy are not solely based on udder health assessments related to the presence of pathological conditions (such as clinical or subclinical mastitis), but also on the economic impact associated with these disorders [98]. As entrepreneurs, farmers aim to optimize the financial performance of their operations, which makes economic considerations a significant factor in their decision-making processes [99]. From a theoretical perspective, SDCT can provide economic benefits by reducing the costs associated with medication use during this stage. However, for this strategy to generate a net positive financial effect, the cost savings must be substantial enough to cover the additional expenses linked to the selective treatment process (e.g., milk sampling and analysis) [100]. Moreover, the inadequate implementation of SDCT, such as failing to use ITSs, can lead to an increased incidence of clinical and subclinical mastitis in subsequent lactations [32,33], potentially resulting in significant economic losses [101]. These losses are typically associated with higher treatment costs, reduced milk production, and the premature culling of affected animals [102].

The scientific literature includes relatively few studies that provide a detailed analysis of the economic impact of SDCT, as the cost-effectiveness of this approach depends on multiple factors, including the accuracy of diagnostic tests, labor costs, antibiotic prices, and the prevalence of IMIs at the time of drying off [19]. For example, in a financial impact analysis of a quarter-level culture-guided SDCT program implemented on a U.S. dairy farm, the cost reduction (USD 9.50 per cow) due to decreased antibiotic use (48%) offset the additional costs associated with on-farm rapid culture systems (USD −6.73 per cow), resulting in a net positive economic impact of USD 2.77 per cow [103]. In a broader study, Rowe et al. [100] compared the economic impact of BDCT with two alternative approaches: culture-guided SDCT and algorithm-guided SDCT, using both deterministic and stochastic budget analyses. The main conclusions of the study indicated that algorithm-guided SDCT is consistently more cost-effective than culture-guided SDCT, and both methods are more economical than BDCT. This economic advantage is primarily due to the lower implementation costs of the algorithm-based approach, which provides a greater capacity to offset potential financial losses associated with a rise in mastitis incidence. Moreover, the same study demonstrated that, in scenarios where antibiotic use was reduced to 20% or 40%, SDCT remained profitable, even with up to a 2% increase in mastitis incidence, suggesting that, in certain contexts, algorithm-based SDCT may be more economically advantageous than BDCT, even in the face of moderate udder health deterioration post-calving. Le Page et al. [104] further supported these findings, emphasizing the critical role of ITSs within SCC-guided SDCT protocols. Their research indicated that using SCC as the sole indicator without ITSs is only profitable up to a threshold of 100,000 cells/mL; beyond this level, SDCT protocols become less cost-effective than BDCT. Conversely, an SCC-based SDCT protocol with a threshold of 200,000 cells/mL, combined with ITSs for all cows, generated an additional economic benefit of CAD 12.13 per dry cow compared to BDCT.

It has also been observed that the optimal proportion of cows requiring antibiotic treatment at drying off is influenced by market fluctuations. For instance, when milk prices are high and mastitis-related costs are substantial, using a higher proportion of antibiotics can be economically justified to prevent production losses. Conversely, lower milk prices may favor reduced antibiotic use [105]. While SDCT may not always generate significant economic benefits for large herds, the available studies suggest that its use does not negatively impact the overall economic balance, contributing to reduced antibiotic consumption without substantially increasing overall costs [27,105].

Therefore, according to the available research, prudent antibiotic use at dry-off can be an economically viable option. However, given the significant variability in SCC levels, IMI prevalence, and MY across farms, SDCT recommendations should be specifically tailored to each herd’s circumstances.

## 7. Influential Factors in Udder Health and SDCT Outcomes

The dry period represents a critical phase in the lactational cycle of dairy cattle, during which udder health is influenced by a complex interplay of interdependent factors at the farm and cow levels. These factors include farm management practices, hygiene, infection prevalence, weaning methods, as well as individual physiological characteristics such as age, parity, and milk yield. Additionally, local factors like the formation of the keratin plug and teat integrity play a crucial role in protecting the udder against infections. While certain farm-level management conditions can be modified to reduce the risk of IMIs, physiological factors such as MY and parity are more challenging to control, and their interaction with prophylactic treatments, including SDCT, can significantly impact therapeutic efficacy [23].

### 7.1. Cow-Level Factors

MY prior to drying off is a significant risk factor for the development of IMIs during the dry period and can influence the effectiveness of SDCT. While some studies have not found a significant association between high MY at dry-off and post-calving udder health parameters [106,107], a substantial body of research supports the opposite view. For instance, Rindsig et al. [1] demonstrated that cows left untreated under SDCT protocols, with either extremely high (>9 kg/day) or low (<2 kg/day) MY, have a greater risk of developing IMIs compared to cows with moderate MY. Similarly, recent studies have highlighted a correlation between SDCT outcomes and SCC at the first test in the subsequent lactation, depending on milk yield (MY) at the final test before dry-off [20]. Specifically, cows producing more than 20 kg of milk per day in the last 30 days before dry-off had an SCC that was 30,000 cells/mL higher than cows producing less than 10 kg/day [108,109]. In the same context, Rajala-Schultz et al. [110] observed that, for every 5 kg increase in MY at dry-off beyond a threshold of 12.5 kg/day, the risk of IMI caused by environmental pathogens increased by 77%. One possible explanation for these findings is that cows with high milk yields may experience an insufficient formation of the keratin plug, making them more susceptible to milk leakage after drying off, thereby increasing their vulnerability to new IMI.

Other physiological characteristics of cows that have been identified in the literature as risk factors for udder health include parity and age. High parity is associated with an elevated risk of IMIs during the dry period [111], with some studies concluding that quarters in cows beyond the fourth lactation were more than four times more likely to develop CM within the first 120 days of the subsequent lactation, compared to quarters in cows in their second lactation [66].

Moreover, given that new IMIs typically occur when pathogens breach the teat canal, several studies have identified quarter-level risk factors. According to Biggs [23], assessing teat end condition in late-lactation cows approaching dry-off is as critical as monitoring high-producing cows. The formation of the keratin plug after dry-off is a complex process influenced by individual factors such as high milk flow rates and the composition of the mammary microbiome. For instance, Corynebacterium bovis may exert a protective, competitive effect against major pathogens, but its keratolytic activity may delay keratin plug formation, with potentially uncertain effects on new IMI rates [23]. However, there is limited research on this topic, and it should be further investigated. Additionally, some authors who have investigated inter-quarter dependencies in the context of IMIs and CM have suggested that posterior quarters may be more susceptible to infection [12,112]. However, these findings are based on a limited number of studies, and more recent research incorporating ITSs in their protocols has not confirmed this pattern [4,80].

### 7.2. Farm-Level Factors

A management factor with significant implications for udder health and productivity is the duration of the dry period. Traditionally, the standard dry period is considered to be between 6 and 8 weeks, a timeframe that allows the mammary gland to undergo the processes of involution and tissue regeneration [113]. However, numerous studies have explored the impact of varying dry period lengths on the risk of new IMIs, bacteriological cure rates, and subsequent lactation performance. For instance, some findings suggest that cows receiving conventional antibiotic treatment with dry periods of 60 days or less experience lower rates of new infections and reduced SCC compared to cows with extended dry periods, implying that excessively prolonged dry periods may extend beyond the protective window of residual antimicrobial concentrations, reducing their prophylactic efficacy [1,20]. In contrast, other researchers have advocated for even shorter dry periods, as brief as 30 days [106]. Supporting these findings, Robert et al. [48] reported that cows not treated with antibiotics during the dry period, but with durations longer than 65 days, had a 1.6-fold higher risk of developing new infections in the subsequent lactation compared to cows with shorter dry periods. Conversely, Zecconi et al. [114] demonstrated that dry periods shorter than 45 days may negatively impact the resolution of pre-existing IMIs, reducing bacteriological cure rates by 29% to 41%. Given the conflicting findings in the current literature, further research is needed to establish an optimal dry period length that balances the complete healing of existing IMIs with the effective prevention of new ones, ensuring both udder health and productivity in the subsequent lactation.

Regarding lactation cessation methods, abrupt drying off is a commonly used practice in Italy [21], offering the advantage of easier implementation, particularly in large-scale herds. However, gradual drying off, achieved by progressively reducing milking frequency, significantly lowers milk production prior to the dry period, thereby accelerating mammary gland involution and enhancing the development of natural udder defense mechanisms [93]. Among respondents, there appears to be a consensus that gradually reducing MY before the dry period, combined with maintaining high hygiene standards, is a critical factor for the successful implementation of SDCT [29].

Other management practices reported to significantly impact udder health during the dry period include the use of surgical alcohol for teat disinfection prior to dry-off treatment, the use of chopped straw as bedding for dry cows, and the provision of increased bedding in the weeks leading up to calving, with these measures being associated with lower SCC at the onset of the subsequent lactation [108].

### 7.3. Other Factors

An additional factor influencing the risk of IMIs around calving is the season. Quarters in cows that calved during the summer and autumn have been found to be more susceptible to post-calving IMIs compared to those in cows that calved in winter [12]. Moreover, the season in which lactation is discontinued significantly affects udder health during the dry period [3,111]. The high temperatures and increased humidity typical of summer and early autumn can promote higher pathogen loads in the environment [115,116], while heat stress can lead to immunosuppression, increasing the susceptibility of cows to infections [107,115]. Additionally, differences in housing and nutrition during warmer months may further contribute to this seasonal effect [107,117].

Of course, the success of SDCT largely depends on how well farmers follow veterinary recommendations. A study by Guadagnini et al. [45] carried out on 11 farms in Italy found that 21% of cows did not receive proper treatment. These cows were 3.77 times more likely to develop subclinical mastitis compared to those treated correctly. This shows the importance of providing farmers with better education and regularly monitoring how SDCT is applied. Good communication and cooperation between veterinarians and farmers are essential for effective implementation. In addition, cow-related factors and overall farm management play a key role in preventing and controlling mastitis. As BDCT is being phased out, proper milking practices and careful management during the dry period are becoming even more important to keep infection rates and somatic cell counts low. Therefore, cow-level factors, along with management practices, are critical elements in the prevention and control of mastitis in dairy herds. In the context of phasing out BDCT, management measures implemented during milking and throughout the dry period become even more important for maintaining low IMI and SCC levels.

## 8. Application of SDCT in Other Species

Although data from the scientific literature regarding the application of Selective Dry Therapy (SDT) in species other than cattle are limited, some studies have shown that this strategy can be successfully adapted to sheep and goats, yielding results comparable to those observed in cows.

In small ruminants, subclinical IMIs are associated with increased SCC, severe damage to mammary tissue, significant milk production losses, and a high tendency for infection persistence across lactations [118,119]. These factors justify the use of dry therapy (DT) as a means of effective mastitis control. In meat-producing sheep, Blanket Dry Therapy (BDT) has been associated with reduced IMIs and improved lamb growth. [120]. In dairy sheep, however, evidence on BDT efficacy is limited, and data on SDT are even scarcer [121]. A relevant example is a study conducted on Churra ewes comparing BDT, SDT, and no treatment showed a marked reduction in infection prevalence in both treated groups (18.8% with BDT and 15.6% with SDT) versus control (48.3%), suggesting SDT may be as effective as BDT in improving udder health and milk production [122]. In goats, SDT using cephapirin benzathine on infected udder halves resulted in similarly low new infection rates in the treated (6.7%) and untreated (4.2%) groups, indicating limited preventive benefit of routine antibiotic use in this species, in contrast to findings in cattle [123]. However, the effective implementation of SDT in small ruminants presents specific methodological challenges, especially in accurately identifying infected glands. SCC-based tests, though commonly used in cattle [77,124], show reduced sensitivity in sheep and even more so in goats. In goats, apocrine milk secretion naturally leads to elevated SCC values, even in the absence of infection [125]. Moreover, elevated SCC is often observed in the uninfected halves of goats with IMIs in the opposite gland. Therefore, the bacteriological culture of milk samples remains the gold standard for diagnosing IMIs in sheep and goats [123]. Additionally, SDT should be applied only if the cost of detecting infected glands is lower than BDT. Due to limited research, further studies are needed to confirm its efficacy, improve diagnostics, evaluate cost-effectiveness, and develop species-specific protocols for sheep and goats.

## 9. Conclusions

Based on the current literature, SDCT can reduce antibiotic use without significantly compromising udder health, provided that strict selection criteria are applied and internal teat sealants are routinely used. However, the effectiveness of SDCT depends on factors such as the herd size, individual cow characteristics, management practices, and regional pathogen variation, which means protocols must be tailored to each farm’s specific context. To support the global implementation of SDCT, future research should validate these protocols under different climates, herd sizes, and management systems. Additionally, there is a need for a better understanding and optimization of the selection criteria used by farmers to ensure a balance between accuracy and economic feasibility.

## Figures and Tables

**Table 1:** Summary of reported sensitivity and specificity for IMI detection at dry-off using SCC thresholds.

Bibliographic References	Method	Sensitivity (%)	Specificity (%)
Torres et al. [44]	Three-month SCC recording		
	SCC < 200,000 cells/mL and no CM in the last 3 months or <100,000 cells/mL if there has been CM in the last 3 months. SCC < 100,000 cells/mL and no CM in the last 3 months SCC < 200,000 cells/mL and no CM in the last 3 months SCC < 300,000 cells/mL and no CM in the last 3 months	69.7 (62.9–75.9) 85.1 (79.5–89.6) 71.2 (64.5–77.2) 62.5 (65.5–69.1)	62.4 (57.7–66.9) 34.6 (30.2–39.3) 50.1 (45.3–54.9) 54.4 (49.7–59.2)
Pantoja et al. [66]	SCC from the last monthly test:		
	50,000 cells/mL 100,000 cells/mL 150,000 cells/mL 200,000 cells/mL 250,000 cells/mL 300,000 cells/mL	86 63 51 40 34 30	40 63 69 80 86 88
	SCC at dry-off		
	50,000 cells/mL 100,000 cells/mL 150,000 cells/mL 200,000 cells/mL 250,000 cells/mL 300,000 cells/mL	94 88 76 64 51 49	37 52 60 66 72 76
Kiesner et al. [52]	Three-month SCC recording		
	SCC < 200,000 cells/mL SCC < 100,000 cells/mL SCC < 100,000 cells/mL + CM SCC < 100,000 cells/mL + parity SCC < 100,000 cells/mL + CMT > 1	34.1 (27.8–40.5) 70.5 (64.5–76.7) 72.9 (66.9–78.9) 78.5 (73.0–84.0) 78.5 (73.0–84.0)	94.4 (87.0–100) 80.5 (67.6–93.4) 78.0 (64.2–91.3) 61.0 (45.2–77.0) 50.0 (33.6–66.3)
	SCC from the last monthly test:		
Lipkens et al. [53]	≥50,000 cells/mL ≥100,000 cells/mL ≥150,000 cells/mL ≥200,000 cells/mL ≥250,000 cells/mL ≥500,000 cells/mL	86.0 (82.8–89.3) 68.6 (64.3–72.9) 58.1 (53.5–62.7) 41.9 (37.3–46.5) 36.0 (31.6–40.5) 20.9 (17.1–24.7)	28.7 (24.5–33.0) 52.4 (47.7–57.1) 64.2 (59.8–68.7) 74.4 (70.3–78.4) 79.2 (75.4–82.9) 93.8 (91.6–96.1)
	Geometric mean of the last 3 monthly SCC tests:		
	≥50,000 cells/mL ≥100,000 cells/mL ≥150,000 cells/mL ≥200,000 cells/mL ≥250,000 cells/mL ≥500,000 cells/mL	82.4 (78.8–85.9) 67.1 (62.6–71.5) 49.4 (44.7–54.1) 37.6 (33.1–42.4) 32.9 (28.5–37.4) 12.9 (9.8–16.1)	32.5 (28.1–36.9) 59.5 (54.9–64.1) 71.6 (67.3–75.8) 79.3 (75.5–83.1) 85.3 (82.0–88.7) 95.4 (93.4–97.4)
McDougall et al. [34]	Last SCC result (>108,000 cells/mL) Peak SCC value (>152,000 cells/mL) Mean SCC level (>105,000 cells/mL)	86 82 76	71 74 80
Rowe et al. [31]	>200,000 cells/mL or >2 cases of CM during lactation	72 (57–84)	44 (42–47)

SCC—Somatic Cell Count. CM—Clinical Mastitis. Values in parentheses represent 95% confidence intervals. CMT—California Mastitis Test.

## Data Availability

Data are contained within this paper.
